# Human Recombinant Lactoferrin Promotes Differentiation and Calcification on MC3T3-E1 Cells

**DOI:** 10.3390/pharmaceutics15010060

**Published:** 2022-12-25

**Authors:** Daichi Nagashima, Yukiko Ishibashi, Sachiko Kawaguchi, Megumi Furukawa, Masahiro Toho, Megumi Ohno, Takeaki Nitto, Nobuo Izumo

**Affiliations:** 1 General Health Medical Research Center, Yokohama University of Pharmacy, Yokohama 245-0066, Japan; 2 Pharmaceutical Education Center, Yokohama University of Pharmacy, Yokohama 245-0066, Japan; 3 Department of Biochemistry, Yokohama University of Pharmacy, Yokohama 245-0066, Japan; 4 Laboratory of Pharmacotherapy, Yokohama University of Pharmacy, Yokohama 245-0066, Japan; 5 NRL Pharma, Inc., Kawasaki 213-0012, Japan

**Keywords:** lactoferrin, human recombinant, MC3T3-E1, differentiation, calcification

## Abstract

Lactoferrin (LF), known to be present in mammalian milk, has been reported to promote the proliferation of osteoblasts and suppress bone resorption by affecting osteoclasts. However, the mechanisms underlying the effects of human sources LF on osteoblast differentiation have not yet been elucidated, and almost studies have used LF from bovine sources. The presented study aimed to characterize the molecular mechanisms of bovine lactoferrin (IF-I) and human recombinant lactoferrin (LF-II) on MC3T3-E1 pre-osteoblast cells. MC3T3-E1 cells were treated with LF, ascorbic acid, and β-glycerophosphate (β-GP). Cell proliferation was analyzed using the MTT assay. Alkaline phosphatase activation and osteopontin expression levels were evaluated via cell staining and immunocytochemistry. The differentiation markers were examined using quantitative real-time PCR. The cell viability assay showed the treatment of 100 μg/mL LF significantly increased; however, it was suppressed by the simultaneous treatment of ascorbic acid and β-GP. Alizarin red staining showed that the 100 μg/mL treatment of LF enhanced calcification. Quantitative real-time PCR showed a significant increase in *osterix* expression. The results suggest that treatment with both LFs enhanced MC3T3-E1 cell differentiation and promoted calcification. The mechanisms of calcification suggest that LFs are affected by an increase in *osterix* and *osteocalcin* mRNA levels.

## 1. Introduction

Discovered in 1939, the protein lactoferrin (LF) is present in mammalian milk, secretory fluid, and mature neutrophil granules. It is an 80-kDa iron-binding glycoprotein belonging to the transferrin family that provides nutrition for biological growth [1]. In its apo-type state (free iron ions present), LF binds to Fe^3+^ and/or Cu^2+^, protecting cells and genes from oxidative damage [2]. Additionally, LF inhibits cancer cell growth and has other pharmaceutical benefits such as radioprotective [3,4] and anti-inflammatory effects that improve metabolic disorders and skin conditions [5,6,7]. Research on ovariectomized rats has shown that LF heightens dopamine and serotonin release in the amygdala [8]. Such ovariectomized animal models are commonly used to evaluate skeletal disorders, such as postmenopausal osteoporosis. Over the past 20 years, an increasing number of studies have reported that LF is associated with skeletal development and bone calcification; hence, LF’s commercial availability as a supplement has also increased worldwide [9]. Because bone formation is an important factor in extending healthy life expectancy, LF supplementation can help improve quality of life [10]. Furthermore, research in MC3T3-E1 cells and experimental animals revealed a relationship between LF and vitamin D receptors [11], as well as the protein’s ability to induce osteogenesis via osteoblast proliferation and differentiation [12]. An investigation into the mechanisms of bovine LF, also using MC3T3-E1 cells, found a relationship between the protein kinase A (PKA) and p38 pathways [13]. Although the broad mechanisms of LF-induced bone formation are known, few studies have examined the molecular mechanisms underlying LF’s pharmacological effects on bone calcification and differentiation. The available reports used bovine LF instead of human recombinant LF. Overall, the two types of LF (bovine and human recombinant) have also not been compared, and we hypothesized that human recombinant lactoferrin promotes calcification as well as bovine lactoferrin.

Bone tissue is maintained by homeostasis between osteoblast bone formation and osteoclast bone resorption [14] A lack of bone formation results in osteoporosis [15]. Osteoblast osteogenic processes are well-known and can be classified into three stages: (1) bone proliferation, (2) bone formation, and (3) calcification. Differentiating from mesenchymal stem cells, progenitor osteoblasts proliferate to become mature osteoblasts and produce bone matrix components, such as type I collagen, osteopontin (OPN), and proteoglycans. During differentiation, alkaline phosphatase (ALP) activity is prominent until calcification, when osteocalcin expression increases. Finally, mature osteoblasts are embedded in the calcified bone matrix. Recent data suggest that LF promotes osteoblast proliferation and inhibits bone resorption through acting on osteoclasts [16,17]. Previous studies in steroid-induced osteopenic mice have also reported that LF significantly improves bone quantity [18]. However, the effects of human recombinant LF on osteoblasts have not been clarified. To verify the hypothesis, this study aimed to characterize the molecular mechanisms of bovine (IF-I) and human recombinant (LF-II) LF, using cell viability assays, morphological analysis, and gene expression evaluation to investigate calcification, proliferation, and osteogenesis.

## 2. Materials and Methods

### 2.1. Cell Culture and Materials

MC3T3-E1 cells were purchased from the Riken Cell Bank. Cells were grown in α-minimal essential medium (α-MEM) containing 10% fetal bovine serum (FBS). Bovine or human recombinant LF obtained from NRL Pharma (Kanagawa, Japan) were dissolved in distilled water (Table 1).

### 2.2. Cell Viability Assay

MC3T3-E1 cells were seeded at a density of 1 × 10^4^ cells/well in 96-well plates and cultured in α-MEM supplemented with 10% FBS. After incubation for 1 d, the growth medium was changed to include 100 μg/mL LF (IF-I and LF-II) with or without 50 μg/mL ascorbic acid and 10 mM β-glycerophosphate (β-GP), then incubated for 24 h. Concentration of LF were determined according to previous studies [17]. The 3-(4,5-dimethylthiazol-2-yl)-2,5-diphenyltetrazolium bromide (MTT) assay was performed using a CellQuanti-MTT cell viability assay kit (BioAssay Systems, CA, USA), and absorbance was measured at 570 nm.

### 2.3. Calcification Assay

**Figure 1:**
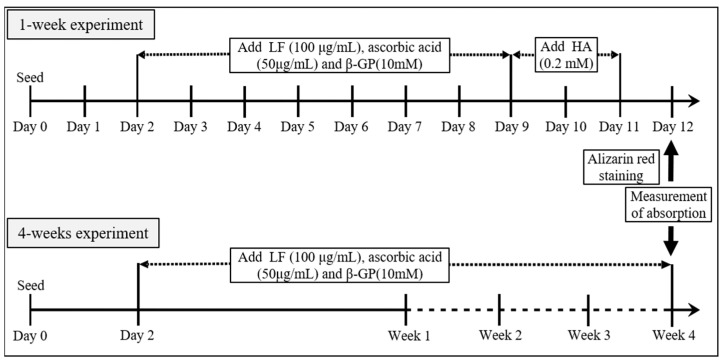
Schedule of alizarin red staining in the 1- and 4-week experimental designs. The MC3T3-E1 cells were seeded, and 100 μg/mL IF-I and LF-II treatments were added along with ascorbic acid (50 μg/mL) and β-GP (10 mM) after 2 d. Absorbance was measured at 1 week of incubation with HA (0.2 mM) treatment, and after 4 weeks of incubation without HA. LF, lactoferrin; HA, hydroxyapatite; β-GP, β-glycerophosphate.

The MC3T3-E1 cells were seeded at a density of 1 × 10^4^ cells/well in 24-well plates. After 2 d of pre-incubation, the growth medium was changed to 2% FBS-containing medium (for the 1-week experiment) or 10% FBS-containing medium (for the 4-week experiment). Next, 100 μg/mL IF-I and LF-II treatments were added, along with ascorbic acid (50 μg/mL) and β-GP (10 mM). For the 1-week experiment, 0.2 mM hydroxyapatite (HA) was also added. After incubation for 2 d, calcification was measured with the addition of 40 mM alizarin red, while the results of cetylpyridinium chloride (CPC) staining were determined via absorbance. The 4-week experiment followed the same procedures, except without HA (Figure 1).

### 2.4. ALP Activity Assay

The MC3T3-E1 cells were seeded at a density of 1 × 10^4^ cells/well in 24-well plates and cultured in α-MEM supplemented with 10% FBS. After incubation for 2 d, the growth medium was changed to a medium containing 2% FBS, 100 μg/mL LF, 50 μg/mL ascorbic acid, and 10 mM β-GP. After culturing for 2 weeks, ALP activity was measured using an alkaline phosphatase detection kit (Sigma-Aldrich, Inc., St. Louis, MO, USA) according to the procedure supplied by the manufacturer, and the number of ALP-positive cells was counted in four random fields per well (n = 6 in each group).

### 2.5. Immunocytochemical Staining

MC3T3-E1 cells were seeded at a density of 8 × 10^4^ cells/well on SPL cell-culture slides and cultured in α-MEM-containing 10% FBS. After incubation for 2 d, the growth medium was changed to 2% FBS; each concentration of IF-I (100 μg/mL) or LF-II (10 μg/mL, 30 μg/mL, and 100 μg/mL), 50 μg/mL ascorbic acid, and 10 mM β-GP-containing medium was differentiated. Cells were cultured for 2 weeks, then stained with anti-OPN primary antibody (R&D Systems, Inc., Minneapolis, MN, USA) and anti-goat IgG antibodies (Sigma-Aldrich Co., LLC., St. Louis, MO, USA). Fluorescence microscopy was used to observe OPN expression.

### 2.6. RNA Isolation and Real-Time Quantitative PCR (RT-qPCR)

MC3T3-E1 cells were seeded at a density of 2 × 10^5^ cells/100 mm dish and cultured in α-MEM-containing 10% FBS. After incubation for 2 d, the growth medium was changed to 2% FBS, 100 μg/mL LF-II, 50 μg/mL ascorbic acid, and 10 mM β-GP. Cells were cultured for 1 week before 0.2 mM HA was added for 2 or 8 h. Next, total RNA was extracted using the SuperScript III first-strand synthesis system for RT-qPCR (Invitrogen, CA, USA). Osteocalcin, runx2, and osterix mRNA expression were assessed using a LightCycler 96 system (Roche Diagnostics K.K., Tokyo, Japan). Their relative expression was normalized to β-actin as the housekeeping gene. The oligonucleotide primers for the amplification of related genes were designed and checked using Primer-BLAST (NCBI, NIH, MD, USA), as listed in Table 2.

### 2.7. Statistical Analysis

Data are expressed as means ± standard deviation (SD). Multiple groups and their corresponding controls in each experiment were compared using one-way analysis of variance (ANOVA), followed by Fisher’s PLSD test. Statistical analyses were performed in Stat View (version 5.0; SAS Institute, Cary, NC, USA). Differences were considered statistically significant at probability (*p*) values < 0.05.

## 3. Results

### 3.1. Effect of LF on Osteoblast Proliferation in Two Types of Media

**Figure 2:**
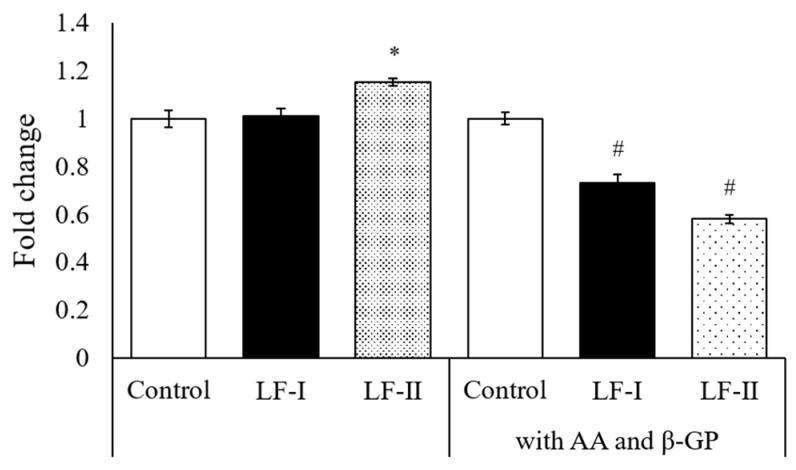
Results of both LFs treatment on MC3T3-E1 cells viability measured by MTT assay. The effects of LF were examined under two experimental set-ups: 1) without ascorbic acid (AA) and β-GP, 2) with AA (50 μg/mL) and β-GP (10 mM). Data are means of change ratios ± SD. * *p* < 0.05, compared with the control for set-up 1 (without AA/β-GP). # *p* < 0.05, compared with the control for set-up 2 (+AA/β-GP). LF, lactoferrin; AA, ascorbic acid; β-GP, β-glycerophosphate.

MTT assay was performed to compare the proliferative capacity of cells in two different media: one with ascorbic acid, β-GP, and LF; the other with LF only. The LF-II-treated group had significantly higher absorbance than the control without ascorbic acid and β-GP. However, absorbance significantly decreased in LF-I- and LF-II-treated groups cultured with ascorbic acid and β-GP (Figure 2).

### 3.2. Evaluation of Calcification Using Alizarin Red Staining

**Figure 3:**
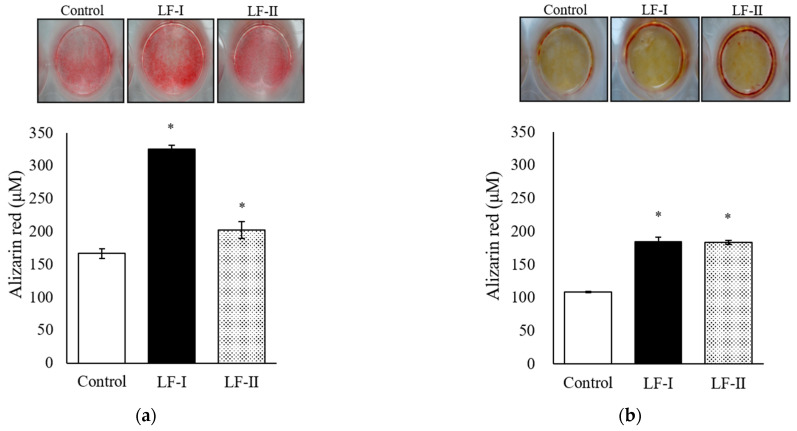
Result of alizarin red staining after 100 μg/mL both LFs treatment with ascorbic acid (50 μg/mL) and β-GP (10 mM) for (**a**) 1 week or (**b**) 4 weeks. (**a**) The 1-week experiment included HA (0.2 mM) for 2 days before alizarin red staining. (**b**) The 4-week experiment was performed without HA. Data are presented as means ± SD. * *p* < 0.05, compared with the control. LF, lactoferrin.

Intensity of alizarin red staining was observed under a light microscope after 1 week of incubation with HA treatment, and after 4 weeks of incubation without HA. Dye intensity increased significantly in LF-I and LF-II versus in the control for both the 1-week and 4-week treatments (Figure 3).

### 3.3. Number of ALP-Positive Cells

**Figure 4:**
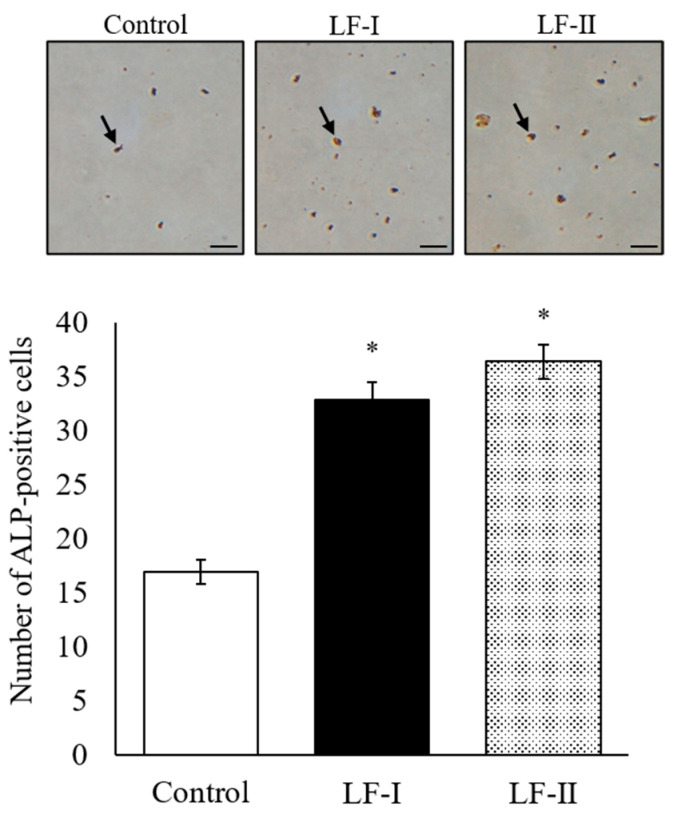
Number of ALP-positive cells after LF treatment with ascorbic acid (50 μg/mL) and β-GP (10 mM) for 2 weeks. Arrows indicate ALP-positive cells, stained red. The number of ALP-positive cells was counted in four random fields per well. Bar = 100 μm; data are presented as means ± SD of six samples in each group; * *p* < 0.05, compared with the control. LF, lactoferrin.

ALP-positive cells were counted under a microscope in both LF-I and LF-II treatment groups after 2 weeks of incubation (Figure 4). The number of activated cells increased in these groups compared to the control. No significant differences were observed between LF-I and LF-II.

### 3.4. Immunocytochemical Staining of OPN Expression

**Figure 5:**
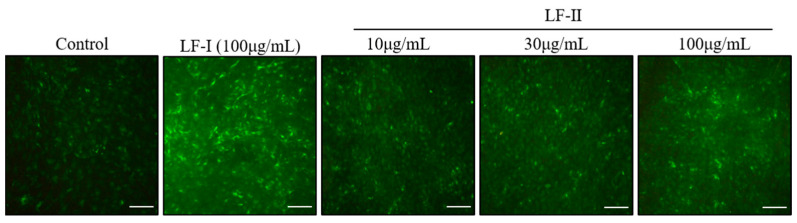
Results of immunocytochemistry after LF-I or LF-II treatment with ascorbic acid (50 μg/mL) and β-GP (10 mM) for 2 weeks. Green-fluorescence intensity shows OPN expression levels. Bar = 200 μm.

After 2 weeks of incubation with LF-I or LF-II, OPN expression was determined via immunocytochemical staining. The LF-I and LF-II group had stronger green-fluorescence intensity than the control group (Figure 5). Moreover, OPN expression increased in an LF-II concentration-dependent trend.

### 3.5. Osteocalcin, Runx2, and Osterix Expression with RT-qPCR

**Figure 6:**
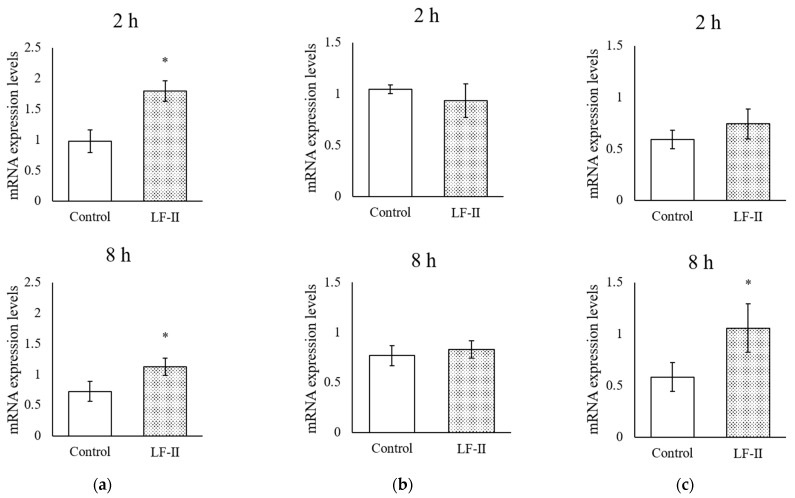
Real-time quantitative PCR measuring mRNA expression levels of bone differentiation and calcification markers, (**a**) *osteocalcin*, (**b**) *runx2*, and (**c**) *osterix*, after LF-II treatment with ascorbic acid (50 μg/mL) and β-GP (10 mM) for 1 week. Total RNA was isolated 2 or 8 h after HA treatment. Data are presented as means ± SD; * *p* < 0.05, compared with the control. LF, lactoferrin.

After incubation with LF-II for 1 week, HA was added for 2 or 8 h before mRNA was isolated to determine the expression of osteocalcin, runx2, and osterix levels (Figure 5). *Osteocalcin* expression increased significantly from control levels after HA addition at both 2 and 8 h (Figure 6). *Osterix* levels did not change after 2 h of HA addition, but increased significantly after 8 h (Figure 6). Runx2 levels did not change in either the 2 or 8 h group (Figure 6).

## 4. Discussion

Our hypothesis which human recombinant LF would promote calcification on MT3T3-E1 cells was accepted because of following results; the present study demonstrated that LF treatment for 1 and 4 weeks promoted calcification and significantly decreased cell viability following addition of 50 μg/mL ascorbic acid and 10 mM β-GP. MTT assay showed that cell viability was significantly increased in the LF-II-treated group without ascorbic acid and β-GP. This suggests that ascorbic acid and β-GP act as differentiation-inducing factors in MC3T3-E1 cells, because when these factors were added the cell viability decreased significantly. Naot et al. reported that LF promotes osteoblast proliferation [19]. Thus, LF-I and LF-II may induce cell differentiation. However, to confirm this hypothesis, future studies are needed to validate the cell-differentiation effects of ascorbic acid and β-GP alone.

Alizarin red staining showed that a 1-week LF treatment significantly promoted calcification with HA and a 4-week treatment did the same without HA. Bone calcification is caused by the deposition of HA on analogous bone formed from extracellularly secreted collagen fibers. Therefore, MC3T3-E1 cells become mature osteoblasts through LF treatment: 1 week of treatment may produce the bone matrix and calcification from HA incorporation. Moreover, LF treatment for 4 weeks can calcify cells without HA. Thus, LF-I and LF-II may exhibit both calcification and differentiation effects. 

Alkaline phosphatase (ALP) is closely involved in cell differentiation and cell division; it is also involved in calcification via regulating phosphate transport [20]. Because it increases osteoblast differentiation, ALP is used as an osteoblast biomarker [21]. Here, we observed that after 2 weeks of LF-I and LF-II treatment, ALP-positive MC3T3-E1 cells increased significantly over control-group amounts. This result suggests that LF promotes osteoblast differentiation in MC3T3-E1 cells.

Expression of OPN was measured by immunofluorescence. As a member of the integrin family, OPN has Arg-Gly-Asp (RGD) sequences that are vital for cell adhesion. The protein is rapidly incorporated into calcified bone-like nodules upon calcification in osteoblast culture systems and is likely involved in calcified bone growth [22]. Immunocytochemistry studies showed that the LF-II treatment group had greater green-fluorescence intensity than the control group and that the intensity was concentration-dependent trend. Moreover, it was observed strongly at 100 μg/mL LF-II, and was comparable to the same concentration LF-I-treated group. Thus, LF-I and LF-II were involved in calcified bone growth, and increase OPN expression levels may promote the formation of calcified bone-like nodules through increasing osteoblast differentiation indices.

To understand the biological impact of LF-II treatment on MC3T3-E1 cells, mRNA expression levels were determined using quantitative real-time PCR. After 8 h of treatment, we observed significant increases in the expression of *osteocalcin* and *osterix*, both differentiation markers of osteogenesis. In contrast, runx2 expression did not change significantly at any treatment time. The Runx2 protein determines mesenchymal-stem-cell differentiation into pre-osteoblasts [23]. Subsequently, Wnt signaling and osterix trigger pre-osteoblast differentiation into immature osteoblasts that produce bone matrix, while inhibiting their differentiation into chondrocytes [24]. Furthermore, osterix regulates osteocalcin involvement in calcification [24]. These results, supported previous studies which bovine LF improved osteogenic marker genes in vivo and in vitro, suggest that LF-II can affect *osterix* expression and enhance calcification after 2–8 h, demonstrating that human recombinant LF has similar effects as bovine LF [11,12,13,17].

This study has some limitations. First, we did not evaluate all LF treatment outcomes and conditions using ALP activity assays, immunocytochemistry, and RT-qPCR. We focused on calcification-related genes that influenced early cell differentiation and collected data only during specific experimental periods. Further experiments are required to investigate how gene expression may change after long-term (>4 weeks) LF treatment. Second, while we measured *osterix* and *osteocalcin* mRNA expression, we did not investigate whether protein expression changed under LF treatment. The lack of data on LF-I treated conditions is also a limitation of the present study. Therefore, we consider ours to be a preliminary study on calcification mechanisms, and future research should investigate LF-induced protein expression and clarify pathways involved in calcification.

## 5. Conclusions

Treatment with both LFs enhanced MC3T3-E1 cell differentiation and promoted calcification. We hypothesized that LF-II would promote calcification on MC3T3-E1 cells. Partially in line with our hypothesis, we found that treatment of LF-I and LF-II for 2 weeks were significantly increased number of ALP-positive cells, and OPN expression was observed strongly without HA. Moreover, treatment of both LFs for 4 weeks were significantly increased alizarin dye intensity without HA. The mechanisms of calcification were suggested that LF-II treatment for 8 h increased the mRNA levels of *osterix* and *osteocalcin*. These results support previous studies suggesting that calcification mechanisms involve an increase in *osterix* and *osteocalcin* mRNA levels after the bovine LF treatment. We provided empirical evidence demonstrating the effect of bovine and human recombinant LF on osteoblasts.

## Figures and Tables

**Table 1:** Sources of the two types of lactoferrin (LF) used in this study and the purity of the concentrations thereof.

Abbreviation	Source	Purity
LF-I	Bovine	>95%
LF-II	Human recombinant	>95%

**Table 2:** Oligonucleotide primers used for quantitative real-time PCR.

Mouse	Universal Probe Library	Forward Primer	Reverse Primer
Osteocalcin	#32	5′-AGACTCCGGCGCTACCTT-3′	5′-CTCGTCACAAGCAGGGTTAAG-3′
Runx2	#34	5′-GCCCAGGCGTATTTCAGA-3′	5′-TGCCTGGCTCTTCTTACTGAG-3’
Osterix	#106	5′-CTCCTGCAGGCAGTCCTC-3′	5′-GGGAAGGGTGGGTAGTCATT-3′
β-actin	#64	5′-CTAAGGCCAACCGTGAAAAG-3′	5′-ACCAGAGGCATACAGGGACA-3′

## Data Availability

Not applicable.
